# One-way routes complicate cooperation in migrant crises

**DOI:** 10.1038/s41598-021-92861-1

**Published:** 2021-06-29

**Authors:** Alessandro Del Ponte, Peter DeScioli, Aidas Masiliunas, Noah Lim

**Affiliations:** 1 Global Asia Institute, National University of Singapore, Singapore, Singapore; 2 Department of Political Science, Stony Brook University, Stony Brook, USA; 3 Department of Marketing, NUS Business School, National University of Singapore, Singapore, Singapore

**Keywords:** Psychology, Human behaviour

## Abstract

How can countries cooperate to shelter migrants? We use experimental economics to study the distinct challenges of cooperation in migrant crises. We designed an economic game, pass the buck, where participants are leaders of countries who decide whether to shelter migrants or pass them to the next country. We examine the difficulties posed by one-way migration and differences in wealth. We find that leaders sheltered migrants less often when they received them first on a one-way route, compared to when everyone received migrants at the same time. Moreover, the first leader became more likely to shelter migrants when the last country could return them to the first. When one country was wealthier, the wealthy leader sheltered more and the other leaders passed more. We discuss the implications for international cooperation in migrant crises.

## Introduction

‘We can do it!’ exclaimed Chancellor Angela Merkel on August 31, 2015 as she announced that Germany would welcome hundreds of thousands of Syrian refugees^1^. Germany’s announcement showed not only compassion for refugees but also remarkable solidarity with European nations who had struggled with migrant crises. Previously, European governments argued over who was responsible for caring for migrants. As often happens, some governments diverted migrants to neighboring countries to avoid the costs of sheltering them. For instance, governments transported African migrants and Syrian refugees back and forth across European borders, even trespassing the border with the military to enforce deportation^2,3^. Similarly, the US disputed with Mexico over migrants from Honduras^4^ while Thailand, Malaysia, and Indonesia argued over their responsibilities for Burmese and Bangladeshi migrants^5^.

We use experimental economics to study the difficulties of international cooperation in migrant crises. We study participants’ decisions in an economic game designed to model migrant crises. Although we cannot directly study the decisions of governments, we can study the decisions of individuals who share a common psychology with government leaders, including motives for reciprocity and fairness. Moreover, leaders often consider public opinion which is also shaped by the psychology of cooperation. Thus, economic experiments can offer insight into the challenges faced by governments^6–10^.

### Migrant crises and government strategies

Solving a migrant crisis requires cooperation between multiple countries. But migrant crises differ from standard dilemmas such as the prisoner’s dilemma and the public goods game. Due to the constraints of geography, economics, and politics, migrants often follow one-way routes. Countries at the beginning of the route receive most of the migrants and then decide whether to shelter them or pass them to the next country by various means, including by force, encouragements, neglect, and weakening security at the borders. If the first government passes the migrants, then the next government makes the same decision. Migrants may be unable to return to the previous country, creating a one-way route^11^. Thus, the first government decides whether to shelter, and the next governments face the same choice only when the previous government passes.

Of course, governments often refuse to shelter migrants because sheltering is expensive^12,13^ and may anger voters^14^. For instance, in 2017, Italy spent over 4 billion euros to care for migrants^15^. At the peak of the Syrian refugee crisis in 2016, Germany spent 20 billion euros^16^. Governments might try to dodge these costs leading to a tragic game of pass the buck, where each government diverts the costs to other countries which only adds to the total cost of the crisis. When countries pay costs to transport migrants or encourage them to move on, these expenses do not resolve the crisis but only add to the total costs while passing on the responsibility to other countries. Also, diverting migrants can duplicate expenses such as the costs of determining refugee status, screening for health conditions, and providing temporary housing in detention centers. International accords such as the UN Global Compact aim to manage this problem, but they are not binding^17^. Sheltering migrants depends on the goodwill of governments such as Germany in 2015^18^.

The European Union has designed rules to regulate migrant crises. The Dublin Regulation prescribes that the country where migrants first arrive must register and shelter them if they cannot be legally returned to the country of origin^19^. But this *principle of first arrival* is rarely enforced because most migrants arrive at the same southern countries^20,21^, and these countries argue that the principle is unfair^20^. The first countries divert migrants to neighboring countries in various ways, such as failing to register migrants, letting them leave detention centers, and overlooking stowaways on trains^22^. Many migrants travel throughout the EU^23^ and end their journey in the wealthier countries in northern Europe. Overall, this situation harms migrants and adds to the costs for the host countries.

### Reciprocity and fairness

Reciprocity is a common solution to social dilemmas^24–27^. When countries each receive migrants, they could all benefit by sheltering their migrants instead of passing them, provided that their neighbors reciprocate. This would follow the principle of first arrival.

However, when migrants travel on a one-way route, reciprocity is not so easy. Most migrants arrive at the first countries on the route. If these countries shelter, then they cannot expect reciprocity from the countries down the line, since these countries will not receive the migrants. When the next countries do receive migrants, they may blame the first countries for passing them, and so reciprocity prescribes passing as well. Thus, cooperation may be more difficult when migrants travel on one-way routes, because the governments cannot rely on reciprocity.

Cooperation also depends on people’s sense of fairness^28–30^. As we mentioned, countries at the beginning of the route feel that their greater burden is unfair. Furthermore, countries differ in wealth and the resources needed to shelter migrants. Wealthier countries could take more responsibility for migrants like Germany did in 2015, lessening the crisis. But differences in wealth could also make a crisis worse. Countries with less wealth could feel more justified in passing migrants toward wealthier countries, which the wealthier countries might resent.

### The pass-the-buck game

To study one-way routes and wealth in migrant crises, we designed a pass-the-buck game. (The present game is unrelated to a game by Bolle^31^ called “pass-the-buck”.) Three players decide whether to shelter migrants and their choices determine how much money they earn in the study.

Each player represents the leader of a country next to two neighboring countries. Each leader has a budget of $4 to manage migrants. A caravan of migrants reaches the first country on a one-way route. The first leader decides whether to *shelter* them or *pass* them to the second leader. If the first leader shelters, then they pay 3 dollars to care for them and the game ends. If they pass, then they pay 2 dollars to transport the migrants to the second leader, who then decides to *shelter* or *pass* to the third leader. If the second leader passes, then the third leader must pay 3 dollars to shelter the migrants, because they are at the end of the route. Importantly, to model a one-way route a leader can pass migrants only to the next leader and not backward. The leaders have common knowledge of all aspects of the game.

**Figure 1:**
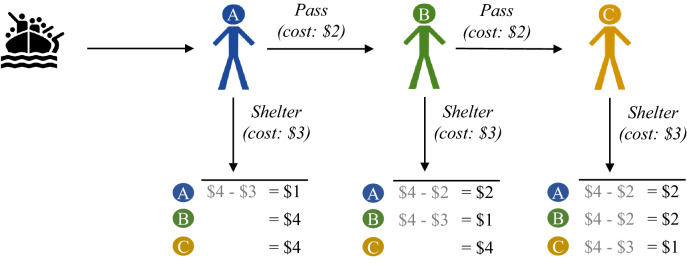
The pass-the-buck game (one-way route).

Figure 1 shows the game. As the payoffs make clear, the leaders face a social dilemma. If the first leader shelters, then the group’s payoffs are $9 in total. But if everyone passes, the group’s payoffs decrease to $5 in total. Particularly, each time a leader passes, they save $1 for themselves while diminishing the group’s payoff by $2 (out of a possible $9, or 22%). Hence, passing is best for the individual but inefficient for the group.

In the following experiments, we study participants’ decisions in the pass-the-buck game. We manipulate elements of the game to test hypotheses based on reciprocity and fairness. We begin by examining whether one-way routes make cooperation more difficult. All experiments were carried out in accordance with the relevant guidelines and regulations under approval from the Institutional Ethics Committee Review Board at the National University of Singapore. Informed consent was obtained from all subjects.

## Experiment 1

To investigate whether one-way routes impede cooperation, we manipulate whether migrants arrive along a one-way route or arrive in all countries. As we discussed, leaders cannot rely on reciprocity when migrants follow a one-way route, because a cooperator would shelter the migrants so the next leader would not make a decision. In contrast, if migrants arrive at all countries, then leaders could reciprocate by sheltering their own migrants while expecting their partners to do the same. Leaders could reciprocate even in a one-shot game if they assume that their partners will cooperate too, as many participants do in experiments on cooperation^25,32–34^. Thus, we test whether participants shelter less when migrants follow a one-way route compared to when they arrive everywhere.

### Methods

We recruited participants from the United States on MTurk^35^. Participants read the instructions and completed a quiz with three questions. We excluded participants with errors on the quiz or incomplete responses (Appendix), yielding a final sample of 200 participants (43% female; age: *M* = 36, *SD* = 11 years). In a between-subject design, participants played a game where migrants arrive either on a one-way route or everywhere symmetrically.

In the one-way condition, participants played the basic game of pass the buck (Fig. 1). Participants read that after matching them with two partners, we would randomly determine the first, second, and third leader in the group. Participants made their decisions as the first and second leader, so we could apply the choice for whichever role they were assigned.

**Figure 2:**
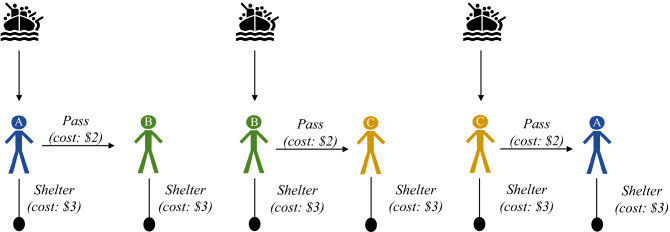
Pass the buck when migrants arrive everywhere.

In the symmetric condition, participants played a similar game except that the migrants arrive everywhere (Fig. 2). Each leader receives a group of migrants. If a leader passes their migrants to the neighbor, then the neighbor must shelter them. The cost to pass ($2) and shelter ($3) are the same as before. To make the final payoffs more comparable across conditions, the leaders had a greater budget of $7, since the leaders had more migrants to shelter and a player could end up sheltering 2 groups. The greater budget holds constant the minimum payoff at $1. Like the original, this game is a social dilemma. Leaders save money by passing but this leads to less payoffs in total. Particularly, each decision to pass reduces the group’s payoff by $2 (out of a possible $12, or 17%).

Importantly, we describe the game to participants with language that fits the subject of migration. For instance, the instructions say that the players represent “governments,” and they decide whether to keep or pass “migrants.” Although it is common to describe games in abstract terms, many experiments use concrete themes, which can increase participants’ comprehension and the psychological realism of the task^36^. Concrete themes add the content and frames that the mind uses to make decisions in a complex world, alongside the quantities and payoffs that they frame. Thus people’s decisions depend on both payoffs and frames, and not only economic frames such as gains and losses^37^ but also social frames such as relationships and morality^36,38–40^. Since framing affects people’s decisions, matching the theme better approximates the dilemmas under study. We also note that the migrant theme is constant across conditions so it could not explain differences between conditions. (It would also be interesting to compare the migrant theme to an abstract description. But we focus on decisions about migration rather than the general issue of framing in economic games.)

After the game, participants explained how they made their decisions and answered questions about their demographics, partisanship, ideology, nationalism, and attitudes toward immigration (Appendix). After the study, we matched participants in groups, determined their role in the game, and calculated their payoffs. Participants earned 50 cents plus 10 cents for each dollar in the game, totaling 77 cents on average (~ 9 min).

### Results and discussion

**Figure 3:**
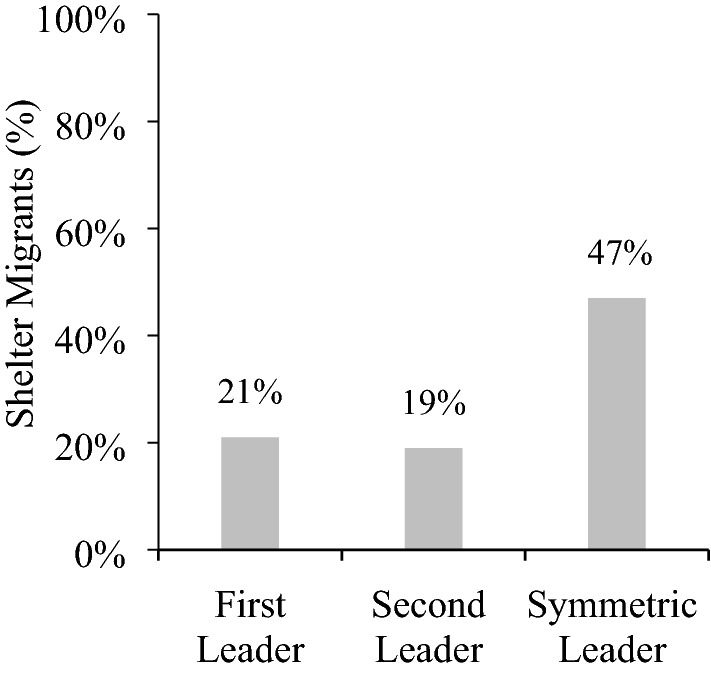
Participants’ decisions to shelter by role.

Figure 3 shows the results. In the one-way route, the first leader sheltered the migrants 21% of the time, cooperating with their group even though they could have earned more for themselves by passing. The other 79% of participants were selfish, passing the buck to the next leader in line. Recall that every decision to pass reduces the group’s payoffs by $2, so this high rate of passing shows that the one-way route makes cooperation more difficult to achieve. The second leader sheltered migrants 19% of the time, which does not differ from the first leader, McNemar’s *χ*^*2*^ (1, *N* = 100) = 0.50, *p* = 0.48.

When migrants arrived everywhere, the symmetric leaders sheltered the migrants 47% of the time. Comparing across games, the first leader sheltered migrants less often than the symmetric leader, *χ*^*2*^ (1, *N* = 200) = 15.06, *p* < 0.001, and the second leader also sheltered less than the symmetric leader, *χ*^*2*^ (1, *N* = 200) = 17.73, *p* < 0.001.

These results support the hypothesis that one-way routes make cooperation more difficult since they exclude reciprocity. On the one-way route, only a fifth of leaders sheltered the migrants, which is less than half as much as when the migrants arrived everywhere.

## Experiment 2

We repeat the same experiment in Singapore. Singapore is a wealthy country surrounded by developing countries so migrants are common. As a result, Singapore has strict policies with stiff penalties for undocumented immigrants and tight requirements for legal migration. Moreover, amid the Covid-19 pandemic, Singaporeans have become more hostile to legal immigration^41^. Influenced by this culture, participants might be even less willing to shelter migrants creating a worse dilemma. But if so, we still expect the one-way route to be more difficult without the possibility of reciprocity.

We recruited 172 undergraduates at the National University of Singapore (59% female; age: *M* = 21, *SD* = 2 years). Participants completed the study online following the same procedures as Experiment 1. Participants earned extra credit plus a bonus of one Singapore dollar per dollar earned in the game. Participants earned an average bonus of $2.60 (~ 13 min).

### Results and discussion

**Figure 4:**
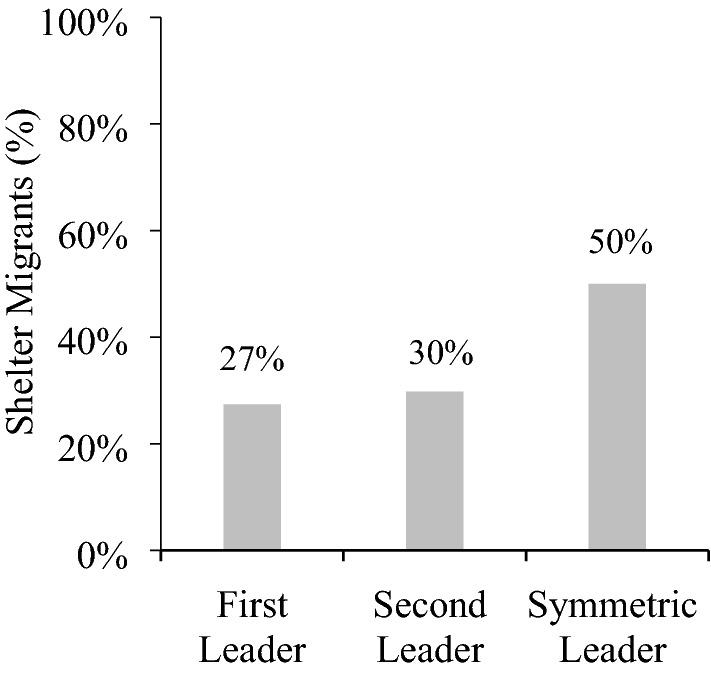
Participants’ decisions to shelter by role.

Figure 4 shows the results. In the one-way route, 27% of the first leaders sheltered the migrants, while the remaining 73% passed them to the next leader. The second leader sheltered the migrants 30% of the time, which does not differ from the first leader, McNemar’s *χ*^2^ (1, *N* = 84) = 0.40, *p* = 0.75.

When migrants arrived everywhere, 50% of the symmetric leaders sheltered the migrants while the other half passed them to the third leader. Comparing across games, the first and second leaders sheltered less than the symmetric leaders, *χ*^*2*^ (1, *N* = 172) = 9.25, *p* < 0.01 and *χ*^*2*^ (1, *N* = 172) = 7.33, *p* < 0.01, respectively.

Thus, in a different culture we find the same experimental difference as before. On the one-way route, participants sheltered migrants less than when migrants arrived everywhere. Generally, despite their strict culture, Singaporean participants were not less likely to shelter than the U.S. participants in Experiment 1 (*χ*^*2*^ test for each role; all *p*s > 0.05).

## Experiment 3

We now look further at the difficulty of one-way routes by comparing to a circular route, where the third leader can return migrants to the first leader.

Like the one-way route, the circular route begins with one leader who receives the migrants, and thus the first leader has an unequal burden. The only difference is that the circular route has an additional leg where the third leader can pass migrants back to the first. Now if the first leader pays to pass the migrants, they risk having to pay again to shelter them if the other leaders pass too. With the added leg, the circular route is no longer a social dilemma. The equilibrium is for the first leader to shelter since they would expect the next leaders to pass, and sheltering is also best for the group.

So rationally the first leader should shelter on the circular route, cooperating more than in the one-way route. If so, this would further detail the challenges of one-way routes. Namely, leaders can shirk without consequences. And it could support policies that return migrants to governments that pass them, such as the Dublin Regulation in which a government can request to return migrants to the country of first arrival.

But the first leader may not shelter as rationality prescribes. They might not look ahead to the end of the game to deduce the equilibrium by backward induction, or they might hope that another leader will shelter despite their self-interest. Moreover, people will sometimes accept losses to reject a situation that they think is unfair. In the ultimatum game, people give up money to reject an unfair deal^42^. If the first leader feels their burden is unfair, they could pass the migrants spitefully even if they know they’ll lose money.

We recruited 200 participants (40% female; age: *M* = 37; *SD* = 11 years) from the United States on MTurk. Participants followed the same procedure as the previous experiments. They were randomly assigned to the game with the one-way route or the circular route.

In the circular route, the third leader could pass the migrants back to the first. And participants started with a budget of $6 so that the first leader’s minimum payoff is still $1 if everyone passes. The one-way route was the same as before except participants had a budget of $6 to match the game with the circular route. Participants earned 50 cents plus an average bonus of 43 cents, totaling 93 cents (~ 9 min).

### Results

**Figure 5:**
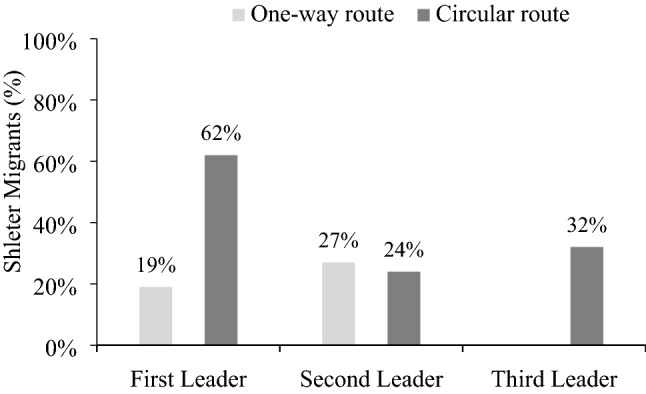
Sheltering by role (first, second, and third leader) and route (one-way or circular).

Figure 5 shows the results. In the one-way route, the first leader sheltered the migrants 19% of the time, and the second leader did so 27% of the time, which is slightly more often, McNemar’s *χ*^*2*^ (1, *N* = 100) = 6.40, *p* < 0.05. Like in the previous experiments, most participants passed the migrants on the one-way route.

Comparing the routes, the first leader sheltered less in the one-way route than the circular route where they sheltered 62% of the time, *χ*^*2*^(1) = 38.37, *p* < 0.001. Thus, participants cooperated less on the one-way route than the circular route, as expected for rational players and again underscoring the difficulty of one-way routes.

Continuing on the circular route, the second leader sheltered the migrants 24% of the time, which is less than the first leader, McNemar’s *χ*^*2*^ (1, *N* = 100) = 38.00, *p* < 0.001. The third leader sheltered migrants 32% of the time, which is also less than the first leader, McNemar’s *χ*^*2*^ (1, *N* = 100) = 18.00, *p* < 0.001, and slightly more than the second leader, McNemar’s *χ*^*2*^ (1, *N* = 100) = 4.57, *p* < 0.05.

### Discussion

We find that the first leader on the one-way route sheltered the migrants only a third as often as on the circular route. When the last leader could return the migrants to the first leader, over 60% of the first leaders sheltered them right away with this deterrent in place. As a result, the groups on the circular route were more efficient. Of course, the remaining 40% of participants still passed despite the rationality of sheltering, presumably due to a lack of foresight or due to spite at the unfair burden. These results suggest that policies that return migrants to the country of first arrival could encourage governments to shelter them.

## Experiment 4

Finally, we examine how cooperation in one-way routes depends on differences in wealth. In addition to unequal burdens in migrant crises, countries often differ in wealth, budgets, and infrastructure to care for migrants, such as the differences between Germany and Italy, or the United States and Mexico.

We compare pass the buck with equal budgets ($4) to three conditions where one leader has a greater budget ($8) than the others. In different conditions, the first, second, or third leader have a budget of $8, representing the rich country relative to the others.

Rationally, the different budgets do not affect the equilibrium. A leader still earns the most by passing. But different budgets could change the leaders’ judgments of fairness. Specifically, the rich leader could be more likely to shelter since they can better afford it. And the other leaders could be more likely to pass toward the rich leader for the same reason.

We recruited 400 participants from the United States on Mturk (41% female; age: *M* = 37; *SD* = 11 years). The procedure was the same as the previous experiments. Participants were assigned to one of four conditions: equal budgets, first rich, second rich, or third rich. The game with equal budgets ($4) was the same as the one-way route in Experiment 1 (Fig. 1). When budgets differed, the rich leader had a budget of $8 while the others had $4. Importantly, the instructions did not label the leaders as “rich” or “poor” but simply said their budgets were $8 or $4. (Adding these labels could also be interesting since they might increase sympathy for the poor leaders^43^.) Participants earned 50 cents and an average bonus of 33 cents, totaling 83 cents (~ 8 min).

### Results

**Figure 6:**
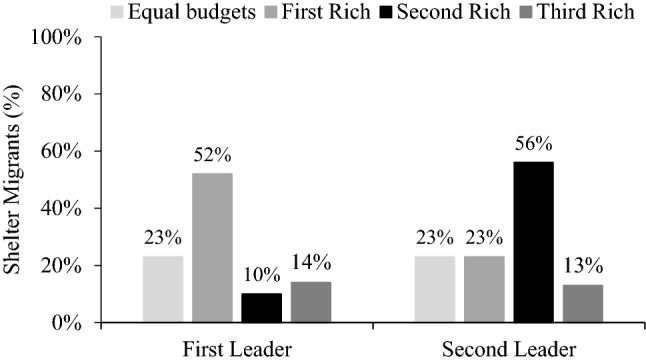
Sheltering by role (first and second leader) and wealth (equal budgets, first rich, second rich, and third rich).

With equal budgets, the first leader and the second leader sheltered the migrants 23% of the time (Fig. 6). So like the previous experiments, most participants passed the migrants.

When one leader had a greater budget than the others, they became more likely to shelter. When the first leader was rich, they sheltered migrants 52% of the time, which is more than double their rate with an equal budget, *χ*^*2*^ (1, *N* = 200) = 17.94, *p* < 0.001. When the second leader was rich, they sheltered 56% of the time, which is also double their rate with an equal budget, *χ*^*2*^ (1, *N* = 200) = 22.78, *p* < 0.001.

We next look at whether poor leaders sheltered less when they could pass to a rich leader. With a smaller budget, the first leader sheltered 10% of the time when the second leader was rich, which is less than when the first leader’s budget was greater than others, *χ*^*2*^ (1, *N* = 200) = 41.23, *p* < 0.001, and equal to others, *χ*^*2*^ (1, *N* = 200) = 6.13, *p* < 0.05. The first leader sheltered 14% when the third leader was rich, meaning the first leader could pass toward the third leader but could only reach them indirectly by burdening the second leader. This rate is less than when their budget was greater than others, *χ*^*2*^ (1, *N* = 200) = 32.65, *p* < 0.001, and only marginally less than with an equal budget, *χ*^*2*^ (1, *N* = 200) = 2.69, *p* = 0.10. Finally, the second leader sheltered 13% when the third leader was rich, which was less than with a greater budget, *χ*^*2*^ (1, *N* = 200) = 40.91, *p* < 0.001, and only marginally less than with an equal budget, *χ*^*2*^ (1, *N* = 200) = 3.39, *p* = 0.07.

Overall, poor leaders sheltered less than rich leaders and sometimes less than equal leaders. Specifically, the first leader sheltered less when they could pass to a rich leader, but the difference was only marginal for the second leader and when the first leader could reach the rich leader only indirectly. We note however that since sheltering was low in the baseline with equal budgets, these marginal decreases may partly reflect a floor effect.

### Discussion

These findings indicate that rich leaders sheltered more than leaders with equal or smaller budgets. But the group’s overall cooperation and efficiency depended on where the rich leader was situated on the one-way route. In fact, when the rich leader was down the line, the other leaders passed the migrants at similar or even higher rates than with equal budgets. Thus, the groups were most efficient when the first leader was rich, whereas leaders passed most of the time when the rich leader was further down the route.

## General discussion

In four experiments, when migrants followed a one-way route, roughly a fifth of the first leaders sheltered the migrants while the majority passed them to the next leader. This observation underscores how difficult it is for governments to cooperate in migrant crises. We further dissected the problem with experimental manipulations. In Experiments 1 and 2, when migrants arrived everywhere symmetrically, roughly half of the first leaders sheltered the migrants, substantially more than the one-way route. This supports the hypothesis that cooperation is more difficult on one-way routes because they preclude the hope of reciprocity, which is a critical foundation for cooperation^44^. Thus, cooperation improved when the migrants arrived everywhere so that leaders could shelter and hope for the same from their neighbors, even though their self-interest was the same and they didn’t know if others in fact cooperated.

In Experiment 3, when the route was circular, meaning migrants could be returned to the first country, most of the first leaders sheltered the migrants right away, three times more than the one-way route. This result confirms that one-way routes are difficult because leaders can pass the burden without consequences. When the migrants could be returned, cooperation improved substantially, indicating that the cooperators looked ahead in the game and rationally responded to the deterrent. The threat of future costs in the circular route is similar to the threat of defection in future rounds of a repeated social dilemma, often called the shadow of the future^44^. Accordingly, the difficulty of cooperation on one-way routes, where shirking lacks future consequences, mirrors the classic finding that cooperation unravels without a shadow of the future. Further, this comparison suggests that leaders would cooperate less when future consequences are uncertain, such as when the regulations that assign responsibilities are not reliably enforced.

In Experiment 4, when the leaders differed in wealth, the rich leader sheltered the migrants over half of the time, which was twice as much as with equal budgets. This result supports the hypothesis that leaders considered fairness in their decisions to shelter, causing them to depart from self-interest which was constant as wealth varied^28–30^. As a result, the group’s efficiency depended on the rich leader’s place along the route. The group was most efficient when the first leader was rich and least efficient when the last leader was rich. Thus, these findings show that one-way routes are further complicated when the wealthier countries are down the line. This consequence of fairness particularly applies to migrant crises in Europe and North America, where migrants arrive at poorer countries such as Greece and Mexico before wealthier countries such as Germany and the United States. Future work could examine other distributions of wealth such as two rich countries and one poor country, along with varying their order on the route. This could help understand more international situations, such as the combination of wealthy countries in Northern Europe relative to neighbors along the migrant routes.

Importantly, the results of these experiments do not automatically generalize to government leaders in a migrant crisis, who must weigh many additional considerations. We use methods from experimental economics to isolate key elements of these problems including unequal burdens, the sequence of decisions, differences in wealth, and the psychology of reciprocity and fairness. Even so, these elements are common in migrant crises, and leaders' decisions are likely to depend on the psychology of reciprocity and fairness as in these experiments, due to their own psychology and their attention to public opinion. However limited, experimental findings add evidence beyond assumptions, like that leaders choose rationally and selfishly, or that they follow a rigid ideology without strategizing and weighing the costs. These common assumptions were falsified in the present experiments, since participants were neither fully selfish nor blindly ideological no matter the incentives and the routes. Thus, the results show that these assumptions about rationality and ideology have limited generalizability even in simplified games.

These experiments also extend previous work on sequential and circular dilemmas, which could offer further insights into migrant crises and other international challenges. In previous experiments on sequential dilemmas, participants contributed more money in the public goods game when their contributions were sequential compared to simultaneous^45–48^. The sequential contributions foster reciprocity because the second player knows whether the first player cooperated, which adds to the first player’s incentive to cooperate. In contrast, when the players decide sequentially whether to pass a burden, reciprocity is precluded: sheltering means the next player does not make a choice so they cannot reciprocate. Accordingly, participants in the present experiments cooperated less in the sequential dilemma, showing that cooperation depends on whether the sequential choices hinder or encourage reciprocity.

Similarly, participants who decided sequentially in a public goods game contributed more money when the first player was rich, starting a cascade of cooperation^49^. In a public goods game with circular choices, participants cooperated more when they could only increase their contributions after observing others’ decisions, applying a commitment mechanism that reinforces reciprocal cooperation^50^. Moreover, in these circular dilemmas, people will pay to find out whether others cooperated, further revealing their reciprocal motives^51^. Finally, the present experiments resonate with research on dilemmas that span generations. Experiments on climate change found that participants created more pollution when it would harm future generations who could not reciprocate^52^.

Understanding the difficulties of migrant crises may also point to solutions. The results of Experiment 3 support policies that return migrants to the countries of first arrival, as prescribed by the Dublin Regulation. But this policy would need to be better enforced to effectively deter passing^21,53^. For example, between 2013 and 2018, out of 188,000 requests to return migrants to Italy where they first arrived, only 24,000 were returned^54^. However, even if enforced, the principle of first arrival may be limited due to differences in wealth and judgments of fairness. One possible solution could be for countries at the end of the route to subsidize the costs of sheltering in the countries of first arrival, which would reduce the unequal burden on those countries.

Without better policies, governments will probably continue to shift the burdens of care to other countries, like most participants did in these experiments. Passing the buck harms migrants and adds to the costs for all countries. The countries of first arrival are in some ways the most burdened. But when leaders pass the burden, the countries at the end of the line and with the most wealth could end up bearing most of the burden. In Europe, for instance, the Scandinavian countries at the end of the route receive large numbers of migrants. More generally, these experiments help to understand governments’ strategies in migrant crises and the distinctive challenges of migrant crises that go beyond the standard dilemmas of cooperation.

## Supplementary Information

Supplementary Information.
