# Supplementary material for: One-way routes complicate cooperation in migrant crises
Source: Sci Rep. 2021 Jun 29;11:13529. doi: 10.1038/s41598-021-92861-1 (PMC8241866; doi:10.1038/s41598-021-92861-1)
Supplement: Supplementary file 1 — Supplementary Information. [file 41598_2021_92861_MOESM1_ESM.docx]

**Supporting Information for**

**One-Way Routes Complicate Cooperation in Migrant Crises**

**Table of Contents**

Supplemental methods for all experiments………………………………………………………..p. 3

Screening of participants…………………………………………………………………………..p. 4

Supplemental results for all experiments………………………………………………………….p. 5

Instructions………………………………………………………………………………………...p. 7

Instructions for Experiment 1 and 2……………………………………………………………….p. 9

Instructions for Experiment 3…………………………………………………………………….p. 10

Instructions for Experiment 4…………………………………………………………………….p. 11

References………………………………………………………………………………………..p. 12

**Supplemental methods for all experiments**

After the game, participants answered a brief questionnaire. We measured attitudes toward immigration asking whether the number of immigrants to America should be reduced, kept the same, or increased (five-point scale from “Reduced a lot” to “Increased a lot”). Moreover, participants answered how strongly they agree or disagree (on a five-point scale) with the following statements: “Immigrants take jobs away.”; “Immigrants are a burden.”; “Immigrants make government services worse.”; “Immigrants undermine American culture.”; “Immigrants are good for America.”; “America should exclude illegal immigrants.”; “Immigrants increase crime rates.” The first and sixth items were reverse-scored. We combined the eight items to form a reliable anti-immigration scale (see Table A2).

To measure nationalism, participants answered how important each of the following is to be American (on a four-point scale ranging from “Not important at all” to “Very important”): “To be born in America”; “To have American citizenship”; “To have lived in America for most of one's life”; “To be able to speak English”. These four items formed a reliable nationalism scale (see Table A2).

Finally, participants reported their age, sex, and answered how strongly or weakly they identify with their party (7-point scale from “very weakly” to “very strongly”), which we coded from 0 (weakest partisan) to 1 (strongest partisan). For ideology, they answered the standard 7-point item (“We hear a lot of talk these days about liberals and conservatives. Here is a 7-point scale on which the political views people might hold are arranged. Where would you place yourself on this scale?”). The scale ranged from “Very liberal” to “Very conservative”.

**Screening of participants**

In recent years, there have been frequent reports about the challenges of ensuring the quality of MTurk samples^1,2^. In line with recommendations in the literature, we used a mix of pre- and post-game screeners to ensure the quality of responses.

After the instructions, we screened participants using a comprehension quiz. The survey terminated if a participant failed to answer a question correctly. Moreover, after making their decision, participants answered an open-ended question where they were asked to write at least 100 characters about how they made their decisions in the game in order for the response to be considered valid. Incomplete answers disqualified the participant.

Comprehension Questions

1. In this game, there are:
   1. 2 players
   2. 3 players
   3. 4 players
   4. 5 players
2. The cost of passing migrants is:
   1. $0
   2. $1
   3. $2
   4. $3
3. The cost of sheltering migrants is:
   1. $0
   2. $1
   3. $2
   4. $3

After the game, participants answered the following open-ended screener:

How did you make your decisions? Please be specific and give us as much information as you can (100 characters minimum).

*Exclusions*

In Study 1, 164 MTurk workers provided incorrect answers on the quiz and thus did not qualify for participating in the game. Moreover, we excluded 111 workers for incomplete responses to the explanation question, yielding the target sample of 200 participants (a 36% post-survey exclusion rate). In Study 2 (students at a university in Singapore), 8 participants failed the comprehension questions whereas no participants provided incomplete responses to the explanation question. In Study 3, 125 workers failed the comprehension quiz while 160 provided incomplete responses to the explanation question (a 44% exclusion rate after the game), yielding the target sample of 200 participants. In Study 4, 277 workers failed the comprehension quiz and 314 failed the explanation question (a 44% exclusion rate), yielding the final sample of 400 participants. These exclusion rates are consistent with figures for post-game or post-survey screeners reported in the literature, which range between 25% and 50%^3–6^.

**Supplemental results for all experiments**

**Table A1**. Demographics and descriptive statistics not reported in the main text.

|  | **Exp. 1** | | **Exp. 2** | | **Exp. 3** | | **Exp. 4** | |
| --- | --- | --- | --- | --- | --- | --- | --- | --- |
|  | **M** | **SD** | **M** | **SD** | **M** | **SD** | **M** | **SD** |
| Partisan strength | 0.65 | 0.27 | - | - | 0.64 | 0.29 | 0.65 | 0.27 |
| Ideology | 0.44 | 0.29 | 0.58 | 0.20 | 0.42 | 0.30 | 0.41 | 0.28 |
| Nationalism | 0.69 | 0.26 | 0.59 | 0.20 | 0.65 | 0.26 | 0.65 | 0.26 |
| Anti-immigration | 0.39 | 0.23 | 0.40 | 0.14 | 0.37 | 0.25 | 0.37 | 0.24 |
| N | 200 | | 172 | | 200 | | 400 | |
| *Note***.** Ideology ranges from 0 (very liberal) to 1 (very conservative). | | | | | | | | |

**Table A2**. Reliability of scales.

|  | Cronbach’s alpha | |
| --- | --- | --- |
|  | U.S. | Singapore |
| Nationalism | 0.84 | 0.62 |
| Anti-Immigration | 0.93 | 0.77 |

| **Table A3**. Zero-order correlations between attitudes, demographics, and sheltering by the first leader across all games (U.S. samples). | | |
| --- | --- | --- |
|  | First leader shelters | |
|  | *r* | *p* |
| Nationalism | -0.13 | < .001 |
| Anti-Immigration | -0.20 | < .001 |
| Republican | -0.04 | .29 |
| Ideology | -0.06 | .09 |
| Age | -0.00 | .89 |
| Female | -0.04 | .31 |
| *Note***.** Ideology ranges from 0 (very liberal) to 1 (very conservative). | | |

**Table A4**. Determinants of sheltering by the first leader across all games (U.S. samples).

|  | (1) | (2) | (3) |
| --- | --- | --- | --- |
| Anti-immigration | -2.35 (0.43) *** | - | -2.23 (0.48) *** |
| Nationalism | - | -1.02 (0.32) *** | -0.20 (0.37) |
| Republican | 0.15 (0.23) | 0.11 (0.23) | 0.16 (0.25) |
| Ideology | 0.39 (0.38) | -0.19 (0.35) | 0.40 (0.38) |
| Age | 0.00 (0.01) | 0.00 (0.01) | 0.00 (0.01) |
| Female | -0.20 (0.16) | -0.19 (0.16) | -0.21 (0.16) |
| Constant | -0.24 (0.29) | -0.02 (0.34) | -0.15 (0.34) |
| *Note*. Logistic regressions with standard errors in parentheses. Higher values of ideology indicate more conservative ideology. N = 800.  * *p* < .05, ** * *p* < .01, *** *p* < .001 | | | |

**Table A5**. Determinants of sheltering by the first leader across all games (Singapore sample).

|  | (1) | (2) | (3) |
| --- | --- | --- | --- |
| Anti-immigration | -1.76 (1.15) | - | -1.78 (1.16) |
| Nationalism | - | 0.08 (0.81) | 0.19 (0.82) |
| Ideology | 0.36 (0.80) | 0.56 (0.79) | 0.38 (0.81) |
| Age | -0.02 (0.08) | -0.02 (0.08) | -0.02 (0.08) |
| Female | 0.35 (0.37) | 0.37 (0.37) | 0.36 (0.37) |
| Constant | 0.24 (1.95) | -0.67 (1.97) | 0.08 (2.07) |
| *Note*. Logistic regressions with standard errors in parentheses. Higher values of ideology indicate more conservative ideology. N = 172.  * *p* < .05, ** * *p* < .01, *** *p* < .001 | | | |

**Table A6**. Correlations between decisions by each leader (U.S. samples).

|  |  | Leader | |
| --- | --- | --- | --- |
|  |  | First | Second |
| One-way | Second | .75 *** |  |
| (8, 4, 4) | Second | .38 *** |  |
| (4, 8, 4) | Second | .16 |  |
| (4, 4, 8) | Second | .87 *** |  |
| Circular | Second | .44 *** |  |
|  | Third | .10 | .67 *** |
| *Note*. * *p* < .05, ** * *p* < .01, *** *p* < .001 | | | |

**Table A7**. Correlation between decisions by each leader (Singapore sample).

|  |  | Leader |
| --- | --- | --- |
|  |  | First |
| Equal budgets | Second | .71 *** |
| *Note*. * *p* < .05, ** * *p* < .01, *** *p* < .001 | | |

**Instructions**

[This part of the instructions is the same for all experiments. The only exception was that in Experiment 3, every participant started with a budget of $6.]

**Overview**

In this study, you can earn money based on your decisions in a game with two other participants. This money is in addition to your $0.50 payment for completing the HIT, which you will earn regardless of what happens in the game.

In the game, you will gain and spend money, and your final balance will determine your MTurk bonus. For each dollar you have at the end of the game, you will receive 10 cents in real money as an MTurk bonus. **To receive this bonus, you must complete the game and the questionnaire that follows.**

Please read the instructions carefully. After the instructions, there will be several comprehension questions, which are easy if you read the instructions carefully. However, if you provide an incorrect answer to the comprehension questions, the survey will terminate, which means that you will be not allowed to submit your decisions and you will receive no compensation (the HIT will be rejected if you submit it).

After the study, **you will be matched with 2 other participants** and everyone’s choices will be carried out to complete the game. The other participants read the same instructions and play the same game as you.

**The Migrant Game**

You and two other participants are the leaders of three countries that span a large island. A group of migrants is headed to the island in three boats after a catastrophe in their homeland.

The leaders need to manage their country’s finances while planning for the migrants. Each leader starts with $4 for managing emergencies.

The migrants arrive at the country ruled by Leader 1. Leader 1 must choose whether to spend money to shelter the migrants or spend money on transportation to pass the migrants to the next country. It costs $3 to shelter the migrants and $2 to pass them.

If Leader 1 pays to shelter the migrants, then the game is over and the other leaders keep their money. If Leader 1 passes the migrants, then they arrive at the second country ruled by Leader 2. Then Leader 2 must make the same decision: Pay $3 to shelter the migrants or pay $2 to pass the migrants.

If Leader 2 shelters the migrants, then the game is over. If Leader 2 passes the migrants, then Leader 3 must pay $3 to shelter them because there are no more countries to pass to.

Please see the following illustration of the game:


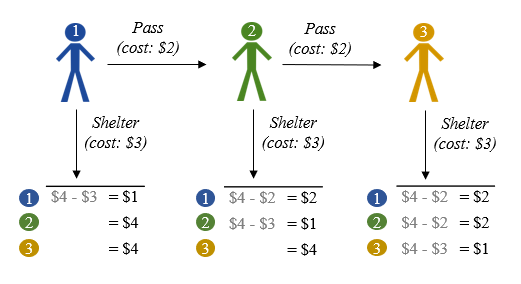


**Illustration of the game.** First, Leader 1 chooses to pass or shelter the migrants. If Leader 1 passes, then Leader 2 chooses to pass or shelter. At the end, the leaders’ earnings are determined by each leaders’ initial funds minus any costs they paid to shelter or pass the migrants.

**Instructions for Experiment 1 and 2**

You and two other participants are the leaders of three countries (Avalon, Beaconia, and Cingutia) that span a large island. A group of migrants is headed to the island in three boats after a catastrophe in their homeland.

The leaders need to manage their country’s finances while planning for the migrants. Each leader starts with $7 for managing emergencies.

Each country gets a boat of migrants at the same time. Each leader makes a single decision: whether to spend money to shelter the migrants or spend money on transportation to pass the migrants to the next country. It costs $3 to shelter the migrants and $2 to pass them.

If a leader pays to pass the migrants, then they arrive to the next country, which must pay to shelter them. So, depending on leaders’ decisions to shelter or pass, a country may end up sheltering one, two, or no boats at all.

Please see the following illustration of the game:


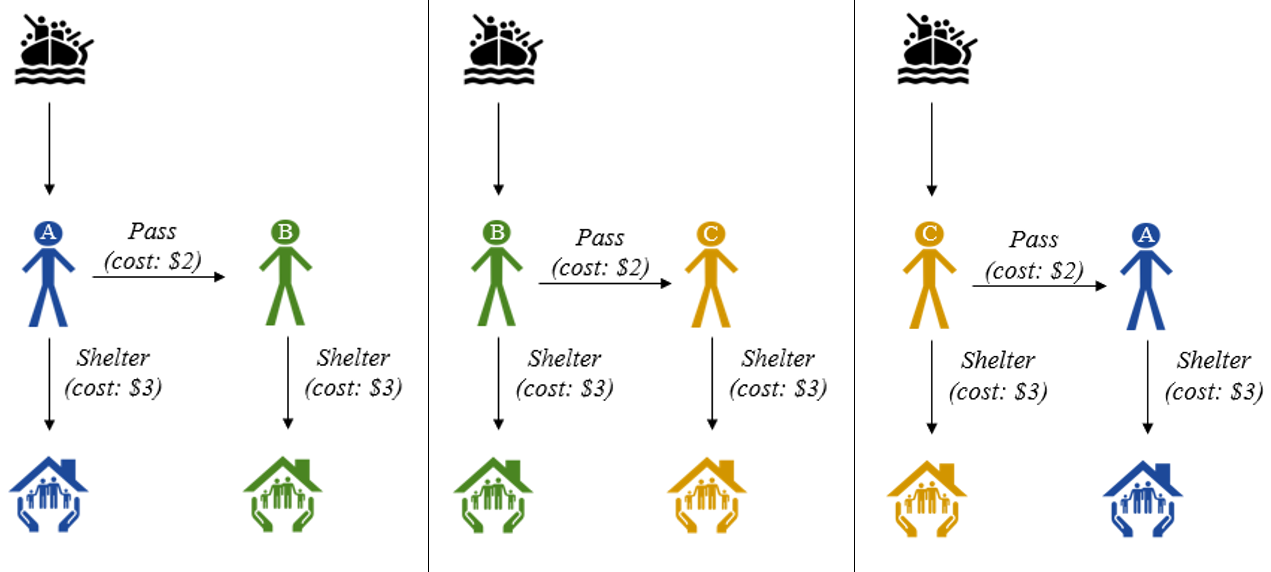


**Illustration of the game.** Each country gets a boat at the same time. A leader can spend $2 to pass the boat to the next country or spend $3 to shelter the migrants. Thus, even if a leader passes their boat, they may get a boat from a neighboring country, in which case they must shelter it. Thus, depending on leaders’ decisions, a country may shelter one, two, or no boats at all.

**Instructions for Experiment 3**

You and two other participants are the leaders of three countries that span a large island. A group of migrants is headed to the island in three boats after a catastrophe in their homeland.

The leaders need to manage their country’s finances while planning for the migrants. Each leader starts with $6 for managing emergencies.

The migrants arrive at the country ruled by Leader 1. Leader 1 must choose whether to spend money to shelter the migrants or spend money on transportation to pass the migrants to the next country. It costs $3 to shelter the migrants and $2 to pass them.

If Leader 1 pays to shelter the migrants, then the game is over and the other leaders keep their money. If Leader 1 passes the migrants, then they arrive at the second country ruled by Leader 2. Then Leader 2 must make the same decision: Pay $3 to shelter the migrants or pay $2 to pass the migrants.

If Leader 2 shelters the migrants, then the game is over. If Leader 2 passes the migrants, then they arrive at the third country ruled by Leader 3. Then Leader 3 must make the same decision: Pay $3 to shelter the migrants or pay $2 to pass the migrants.

If Leader 3 shelters the migrants, then the game is over. If Leader 3 passes the migrants, then they arrive back at the first country ruled by Leader 1. This time, Leader 1 must pay $3 to shelter them because each leader can only pass the migrants once.

Please see the following illustration of the game:


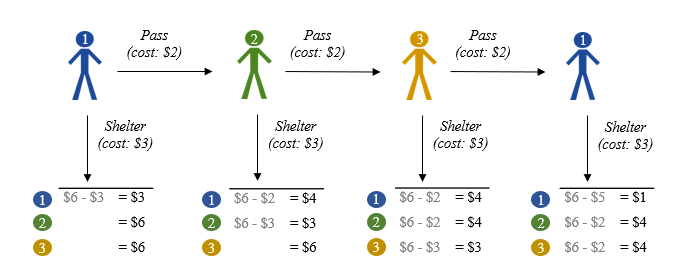


**Illustration of the game.** First, Leader 1 chooses to pass or shelter the migrants. If Leader 1 passes, then Leader 2 chooses to pass or shelter. If Leader 2 passes, then Leader 3 chooses to pass or shelter. If Leader 3 passes, then the migrants return to Leader 1, who must shelter the migrants. At the end, the leaders’ earnings are determined by each leaders’ initial funds minus any costs they paid to shelter or pass the migrants.

**Instructions for Experiment 4**

You and two other participants are the leaders of three countries that span a large island. A group of migrants is headed to the island in three boats after a catastrophe in their homeland.

The leaders need to manage their country’s finances while planning for the migrants. The countries differ in wealth so the leaders begin with different amounts of money for managing emergencies: Leader 1 starts with ($8, $4, $4); Leader 2 starts with ($4, $8, $4); and Leader 3 starts with ($4, $4, $8).

The migrants arrive at the country ruled by Leader 1. Leader 1 must choose whether to spend money to shelter the migrants or spend money on transportation to pass the migrants to the next country. It costs $3 to shelter the migrants and $2 to pass them.

If Leader 1 pays to shelter the migrants, then the game is over and the other leaders keep their money. If Leader 1 passes the migrants, then they arrive at the second country ruled by Leader 2. Then Leader 2 must make the same decision: Pay $3 to shelter the migrants or pay $2 to pass the migrants.

If Leader 2 shelters the migrants, then the game is over. If Leader 2 passes the migrants, then Leader 3 must pay $3 to shelter them because there are no more countries to pass to.

Please see the following illustration of the game:


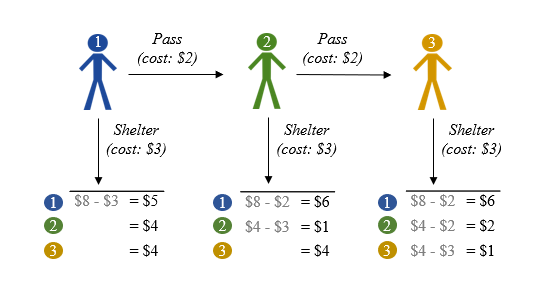


**Illustration of the game.** First, Leader 1 chooses to pass or shelter the migrants. If Leader 1 passes, then Leader 2 chooses to pass or shelter. At the end, the leaders’ earnings are determined by each leaders’ initial funds minus any costs they paid to shelter or pass the migrants.

**References**

1. Chmielewski, M. & Kucker, S. C. An MTurk crisis? Shifts in data quality and the impact on study results. *Social Psychological and Personality Science* **11**, 464–473 (2020).

2. Kennedy, R. *et al.* The shape of and solutions to the MTurk quality crisis. *PSRM* **8**, 614–629 (2020).

3. Berinsky, A. J., Margolis, M. F. & Sances, M. W. Separating the Shirkers from the Workers? Making Sure Respondents Pay Attention on Self-Administered Surveys. *American Journal of Political Science* **58**, 739–753 (2014).

4. Downs, J. S., Holbrook, M. B., Sheng, S. & Cranor, L. F. Are your participants gaming the system? Screening Mechanical Turk workers. in 2399–2402 (2010).

5. Goodman, J. K., Cryder, C. E. & Cheema, A. Data collection in a flat world: The strengths and weaknesses of Mechanical Turk samples. *Journal of Behavioral Decision Making* **26**, 213–224 (2013).

6. Horton, J. J., Rand, D. G. & Zeckhauser, R. J. The online laboratory: Conducting experiments in a real labor market. *Experimental economics* **14**, 399–425 (2011).
